# Preparation and Characterization of Oleogels Based on Cellulose Modified by High-Pressure Microfluidization and Rubber Seed Oil Body

**DOI:** 10.3390/gels11100819

**Published:** 2025-10-13

**Authors:** Zhipeng Meng, Lei Wang, Kai Jiang, Guoqin Liu

**Affiliations:** School of Food Science and Engineering, South China University of Technology, Guangzhou 510641, China; 17596545640@163.com (Z.M.); wanglei.sky.me@163.com (L.W.); jjking2101@163.com (K.J.)

**Keywords:** oil bodies, cellulose, modification, oleogel, rubber seed

## Abstract

This study aimed to minimize the amount of gelator used in oleogel preparation and enhance the valorization of rubber seeds. Cellulose extracted from rubber seed shells was modified via high-pressure microfluidization (HPM), which significantly enhanced its specific surface area from 0.92 m^2^/g (CL) to 6.47 m^2^/g (MCL), along with markedly improved water-holding capacity (WHC increased from 4.92 to 29.37 g/g) and swelling capacity (SC increased from 0.65 to 3.38 mL/g). The modified cellulose (MCL) served as the gelator, while rubber seed oil bodies (OBs), isolated through sucrose-assisted extraction, functioned as the oil phase. A series of OB emulsions containing 0% to 0.75% MCL were prepared and subsequently converted into oleogels by freeze-drying and shearing. Oleogels containing ≥0.45% MCL exhibited excellent oil binding capacity (OBC > 98.6%) and strong gel strength (storage modulus G′ > 10^5^ Pa). Texture profile analysis further confirmed significant improvements in the textural properties of the oleogels with increasing MCL content. These findings demonstrate that MCL, combined with rubber seed OBs, enables the development of high-performance oleogels with minimal gelator requirements. This approach not only reduces gelator usage but also provides a novel strategy for the upcycling of rubber seed shells, offering valuable insights for the design of nutrient-rich functional oleogels.

## 1. Introduction

Oleogelation is a well-recognized and viable strategy to create solid-like gels from liquid oils by forming a three-dimensional oleogelator network that physically traps the oil without altering its chemical composition, offering a healthier substitute for saturated fats without altering the original fatty acid profile of the liquid oil [1,2]. In recent years, oleogels have been widely applied in products such as margarine, ice cream, and meat products [3,4]. A significantly high concentration of oleogelator (>10% in the oleogel) is required to form a stable oleogel structure; otherwise, gelation fails, or the resulting oleogel exhibits extremely low mechanical strength [5]. The high oleogelator demand in conventionally prepared oleogels substantially increases production costs, rendering them commercially unviable for food applications. Consequently, developing low-oleogelator-content oleogels to reduce costs is imperative.

Oil bodies (OBs) are natural oil-in-water emulsions found in plant seeds, serving as micron-sized (0.3–20.0 μm) lipid storage structures abundantly present in oil-rich seeds and fruits [6,7,8]. The core of OBs consists of triacylglycerols, surrounded by a protective layer composed of proteins and phospholipids, which confers considerable physical and chemical stability [9]. Mert et al. [10] first utilized xanthan gum and pectin to stabilize an oil body emulsion, demonstrating that only 1.5% hydrocolloid was sufficient to coat the oil bodies. After drying, this process yielded OBs-based oleogels exhibiting high hardness and elastic modulus values, highlighting the advantage of reduced oleogelator requirement when using oil bodies for oleogel preparation. However, research on preparing oleogels directly from oil bodies remains limited. Current methods for fabricating OBs-based oleogels often necessitate repeated pH adjustments to encapsulate the oleogelator onto the oil body surface, resulting in complex operational procedures [7,11,12].

Cellulose, the most abundant polysaccharide in nature, can be modified through physical, chemical, or biological methods to broaden its applications in the field of oleogelation [13]. Zou et al. [14] utilized nanocellulose to prepare oleogels via an emulsion-templated method. The results demonstrated that the resulting oleogels exhibited a high elastic modulus (1.1 × 10^5^ Pa) and oil binding capacity (OBC > 85%). However, the preparation of nanocellulose typically involves extensive processing steps such as acid hydrolysis, centrifugation, dialysis, and auxiliary treatments like ultrasonication or homogenization, resulting in high costs and significant environmental drawbacks [13]. To minimize the use of harmful chemical reagents like strong acids and bases, more environmentally friendly and cost-effective physical modification methods have attracted researchers’ interest. High-pressure microfluidization (HPM) is an emerging physical processing technology that modifies cellulose through intense shear, cavitation, and turbulent forces. This process effectively disrupts the hydrogen bonding network of cellulose, increases its specific surface area, and exposes more hydroxyl groups without the use of chemical reagents [15]. It is noteworthy that cellulose modified solely by HPM (without chemical derivatization) has been successfully applied in the preparation of Pickering emulsions. However, its application in the construction of oleogels has not yet been reported.

According to statistics from the International Rubber Study Group (IRSG), the global rubber tree (Hevea brasiliensis) cultivation area exceeded 15 million hectares in 2024, yielding an estimated 20.84 million tons of rubber seeds. China ranks as the world’s third-largest natural rubber producer, with an annual rubber seed output of approximately 747,000 tons. However, over 75% of these seeds remain abandoned in rubber plantations without utilization [16,17,18]. Rubber seeds, a by-product of rubber production, contain kernels with an oil content of 40–50% [16,19,20]. The high n-3 polyunsaturated fatty acid content, particularly α-linolenic acid, provides cardiovascular benefits by regulating lipid metabolism, exerting anti-inflammatory effects, and protecting vascular endothelial function. This balanced ratio aligns better with dietary recommendations (2:1 to 5:1) than common vegetable oils like soybean or peanut oil, which typically exhibit higher n-6/n-3 ratios [21,22]. Studies confirm that regular consumption of rubber seed oil helps manage hyperlipidemia and hypertension while providing cardiovascular protection and anti-inflammatory benefits [21,23]. Additionally, rubber seed shells contain abundant cellulose (> 70%), serving as a high-quality cellulose source currently utilized primarily in natural rubber composites [24].

To explore the feasibility of preparing oleogels with low oleogelator content and reduced cost, it is essential to investigate the preparation methods and mechanisms of OBs-based oleogels. Accordingly, this study utilized discarded rubber seed resources from rubber plantations. Cellulose extracted from seed shells was modified via a direct HPM treatment, bypassing complex steps like acid hydrolysis, and served as the gelator. Protein-rich OBs extracted from the seeds acted as the oil phase. Without requiring pH adjustment or other complex steps, a low-oleogelator-content OBs-based oleogel with high protein content and liposoluble accompaniments was prepared directly using an emulsion-templated method. The HPM-modified cellulose was characterized, and the impact of modified cellulose on the gel properties of the OBs-based oleogel was investigated.

## 2. Results and Discussion

### 2.1. Cellulose Characterization

#### 2.1.1. Physicochemical Properties of Cellulose

Water-holding capacity (WHC), a key indicator of cellulose functionality related to its hydration properties, helps prevent food shrinkage and influences food viscosity [25,26]. As evident from Table 1, HPM modification significantly increased (*p* < 0.05) the WHC of shell cellulose. This enhancement is attributed to the enlarged specific surface area and formation of a branched network structure that effectively entraps water molecules. Swelling capacity (SC), which reflects the water-absorption expansion capability of cellulose, is positively correlated with WHC [15]. After HPM modification, more water molecules were confined within the cellulose network, leading to a significant volumetric expansion of the fibrous matrix.

Oil-holding capacity (OHC) is a key parameter for evaluating the fat adsorption ability of cellulose. A high OHC allows cellulose to effectively retain lipids during food processing and can contribute to reduced serum cholesterol levels by absorbing fats in the digestive tract [27]. After HPM modification, the OHC of MCL increased significantly by 2.81 times compared to that of CL. This remarkable improvement suggests that the HPM process creates abundant physical adsorption sites and capillary spaces within the cellulose structure, thereby effectively immobilizing oil through physical entrapment.

#### 2.1.2. XRD Analysis of Cellulose

**Figure 1:**
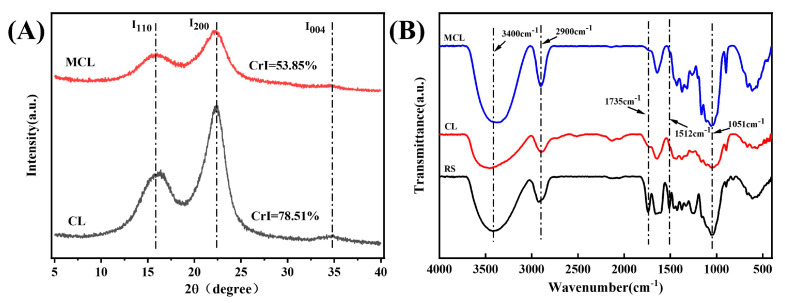
FTIR spectrum (**A**) and XRD pattern (**B**) of the samples. Note: RS, rubber seed shell; CL, native cellulose; MCL, HPM-modified cellulose.

X-ray diffraction (XRD) analysis was employed to investigate changes in cellulose crystallinity, with scans performed over a 2θ range of 5° to 40° to cover the primary characteristic peaks of cellulose [28]. As shown in Figure 1A, both unmodified and modified samples exhibited characteristic diffraction peaks at 2θ = 15.5° (110), 22.5° (200), and 34° (004), confirming that all samples retained the cellulose I (native) crystal structure [26,29]. After HPM modification, the crystallinity index (CrI) decreased significantly from 78.51% to 53.85%, indicating a partial disruption of the crystalline regions. This observation is consistent with the findings of Liu et al. [30], who also reported a reduction in cellulose crystallinity following high-pressure homogenization treatment, demonstrating the efficacy of such physical methods in altering the crystalline structure.

#### 2.1.3. FTIR Analysis of Cellulose

As illustrated in Figure 1B, the broad peak at 3432 cm^−1^ corresponds to O–H stretching vibrations, while the peak at 2900 cm^−1^ is assigned to C-H stretching vibrations [31]. The characteristic absorption peaks observed at 1435 cm^−1^ and 1051 cm^−1^ are ascribed to C-H bending vibrations and C-O-C stretching vibrations, respectively [32]. Lignin and hemicellulose typically exhibit characteristic absorption bands at 1735 cm^−1^ and 1512 cm^−1^. Notably, the peak intensities at 1735 cm^−1^ and 1512 cm^−1^ for CL were significantly reduced, indicating that lignin and hemicellulose were largely removed during chemical treatment, successfully isolating cellulose from the rubber seed shells. After HPM modification, MCL exhibited an FTIR profile very similar to that of CL, suggesting that the modification did not alter the fundamental chemical structure of the cellulose.

#### 2.1.4. Scanning Electron Microscopic of Cellulose

**Figure 2:**
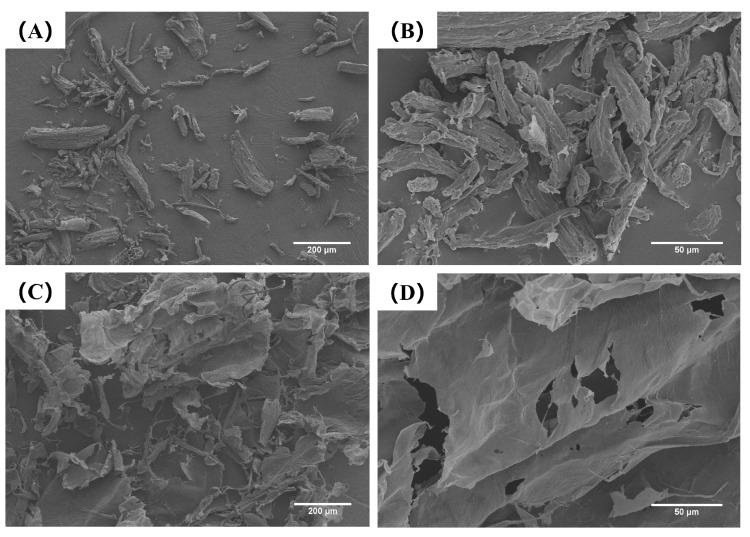
Scanning electron microscopic SEM image of CL (**A**,**B**) and MCL (**C**,**D**). Images were taken at 100× (**A**,**C**) and 500× (**B**,**D**).

As shown in Figure 2, cellulose directly extracted from rubber seed shells (CL) exhibits a block-like or rod-like morphology. After modification by high-pressure microfluidization (HPM), these structures undergo delamination, resulting in sheet-like forms. This morphological transition significantly increased the specific surface area from 0.92 m^2^/g (CL) to 6.47 m^2^/g (MCL). This increase is consistent with the findings of Xie et al. [15]. The expansion in surface area is attributed to the cavitation and high shear forces generated during HPM, which disrupt crystalline domains and induce pronounced swelling and dissociation of cellulose [29]. The exposure of abundant hydroxyl groups through this process appears to facilitate the formation of a denser three-dimensional network via enhanced intermolecular hydrogen bonding. This structural development, supported by the substantially increased surface area, may have contributed to the stabilization of OB emulsions and supported the subsequent formation of OB-based oleogels.

### 2.2. Characterization of OBs-Based Oleogels

#### 2.2.1. Appearance of Freeze-Dried Products and OBs-Based Oleogels

**Figure 3:**
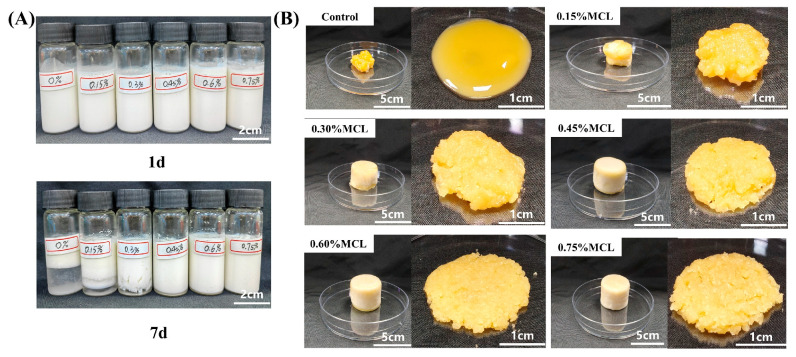
Appearance of OB emulsions (**A**) and lyophilized products of OB emulsions and OB-based oleogels (**B**).

The storage stability of oil body emulsions with varying MCL concentrations (0–0.75%) was evaluated at room temperature prior to lyophilization (Figure 3A). The control sample (without MCL) exhibited poor stability, showing obvious phase separation after 7 d of storage. In contrast, the addition of MCL at concentrations above 0.45% significantly enhanced emulsion stability, preventing phase separation throughout the 7 d period. This improvement is likely attributed to the increased viscosity of the continuous phase, which formed a network structure that effectively retarded droplet movement and coalescence. As shown in Figure 3, the freeze-dried product of OBs without MCL addition collapsed after 48 h of lyophilization. This structural failure occurred because ice crystals formed during pre-freezing punctured the OBs, causing significant oil leakage during lyophilization. Consequently, the sheared OBs-based oleogel exhibited severe oil exudation and failed to form a coherent gel structure. In contrast, when MCL content exceeded 0.45%, the freeze-dried products appeared significantly plumper and firmer, presenting as milky-white solids without surface oil. Li et al. [33] similarly observed that oil exudation on freeze-dried oleogel surfaces gradually diminished with increasing cellulose nanofiber (CNF) concentration (0.4–4%), aligning with our findings. The progressive reduction in surface oil leakage from oleogels prepared with higher MCL content demonstrates effective oil entrapment within the network structure formed by MCL and proteins.

#### 2.2.2. Oil Binding Capacity

The microstructure of the OBs-based oleogels was analyzed by CLSM. As shown in Figure S1, the oleogel prepared without MCL exhibited protein aggregation and severe oil leakage. In contrast, the MCL-structured OBs-based oleogel displayed a continuous three-dimensional network, with proteins closely associated with the cellulose fibrils. This demonstrates that MCL stabilizes the OBs-based oleogel by adsorbing proteins and forming a composite network that effectively immobilizes the oil droplets.

**Figure 4:**
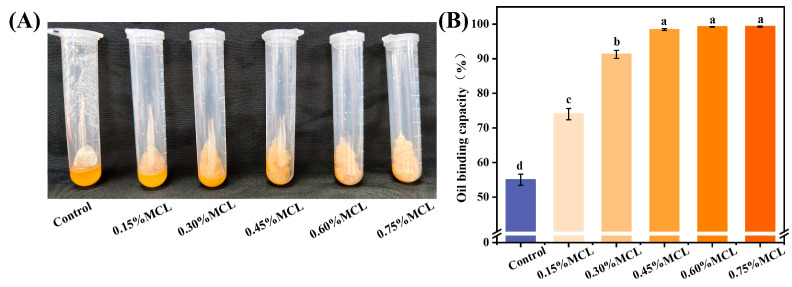
Appearance of OBs-based oleogels after centrifugation (**A**) and oil binding capacity (**B**). Different letters (a–d) indicate significant differences (*p* < 0.05).

As shown in Figure 4B, oleogels prepared solely from OBs exhibited a low oil binding capacity of 55.6%, indicating structural instability. The observed oil leakage suggests that the oil bodies underwent deformation under processing conditions, releasing oil to the surface. In contrast, the incorporation of MCL significantly enhanced the oil binding capacity (*p* < 0.05). This improvement can be attributed to the formation of a composite network in which oil droplets are effectively embedded within the structural framework built by MCL and OB-associated proteins, thereby strengthening the gel’s mechanical resistance to rupture during freeze-drying and shearing. These findings are consistent with those reported by Farooq et al. for freeze-dried emulsions stabilized with chitosan and vanillin [10]. When MCL was added at 0.45% to the OBs emulsion (equivalent to 1.8% in the final oleogel), the resulting oleogel achieved an oil binding capacity of 98.6%. This demonstrates that modified cellulose acts as an effective oleogelator, enhancing oil retention during drying and contributing to a more compact and stable oleogel structure.

#### 2.2.3. Rheological Properties of Oleogels

**Figure 5:**
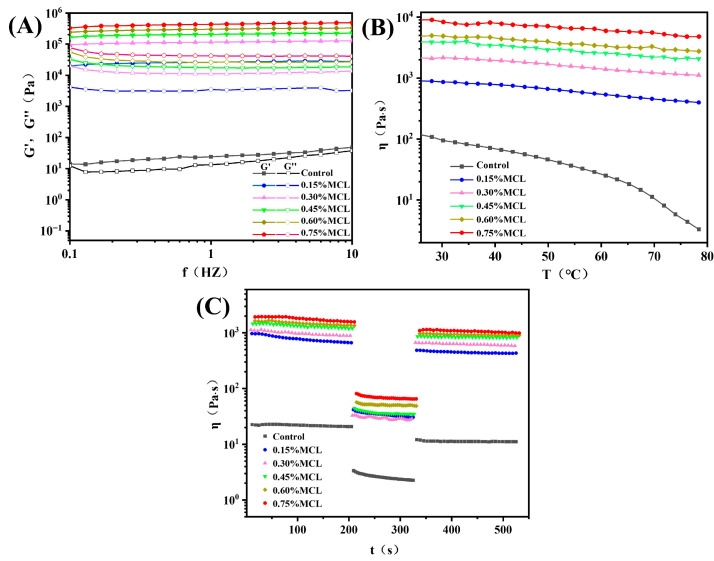
(**A**) Changes in the elastic modulus (G′) and viscous modulus (G″) of oleogels as a function of frequency; (**B**) changes in the apparent viscosity of oleogels as a function of temperature; (**C**) thixotropy test curves of oleogels.

Frequency sweeps evaluate the structural strength and stability of gel networks by monitoring changes in elastic modulus (G′) and viscous modulus (G″) as functions of frequency (f). Prior to frequency sweeps, strain amplitude tests determined the linear viscoelastic region (LVR) of the oleogels. As shown in Figure 5A, all samples exhibited G′ values exceeding G″, confirming solid-like behavior due to restricted oil mobility within the structural framework formed by OB-associated proteins and MCL. Without MCL addition, both G′ and G″ displayed pronounced frequency-dependent increases with rising frequency, indicating potential weak gel formation. However, MCL incorporation significantly increased both moduli at fixed oscillatory frequencies while diminishing frequency dependence. The modified oleogels maintained G′ > G″ without crossover, demonstrating frequency-independent behavior. This reflects strong intermolecular forces binding oil droplets into a relatively stable solid-like network characteristic of “true oleogels” [11]. These observations confirm that the protein-MCL composite network exhibits robust elastic properties.

Temperature sweeps in rheological testing serve as a critical indicator of oleogel thermal stability [34]. As shown in Figure 5B, the flow behavior curve of OBs-based oleogels without MCL exhibited a pronounced decreasing trend. This outcome was anticipated since the microstructure of oil bodies demonstrates poor tolerance to temperature variations, where weakly associated gel networks are readily disrupted. In contrast, MCL-incorporated OB-based oleogels displayed significantly reduced temperature dependence, with minimal viscosity reduction observed. Furthermore, viscosity progressively increased at identical temperatures with higher MCL loading. This behavior confirms that MCL promotes the formation of a denser network structure that effectively encapsulates the oil phase.

The thixotropic behavior of OBs-based oleogels was systematically investigated to evaluate their time-dependent rheological properties. As depicted in Figure 5C, at a constant shear rate of 0.1 s^−1^, the apparent viscosity of the oleogels decreased marginally over time. Samples containing a higher MCL content maintained greater viscosity throughout the shearing process. When the shear rate was increased to 10 s^−1^, all samples exhibited pronounced shear-thinning. Upon returning to 0.1 s^−1^, the deformation recovery ratio (DSR) increased from 51.65% (control) to 63.43% (0.45% MCL). This enhancement is attributed to a strengthened network structure resulting from enhanced intermolecular crosslinking, indicating high thixotropic recovery. This finding is consistent with the study by Roman et al. [35], who reported that increasing the cellulose nanofiber (CNF) content from 0.7 wt% to 1.4 wt% in CNF-based oleogels improved the DSR from 25.5% to 54.1%, alongside a notable reduction in structural breakdown. Although Zou et al. [14] successfully produced oleogels with exceptional thixotropic recovery (DSR > 75%) by optimizing cellulose nanofiber (CNF) diameter, their oil-binding capacity (OBC) was moderate, ranging from 75% to 85%. By comparison, the oleogels structured by MCL and oil bodies in this study demonstrate high structural recovery as well as strong oil-binding capacity.

#### 2.2.4. Texture Profile Analysis of Oleogels

**Figure 6:**
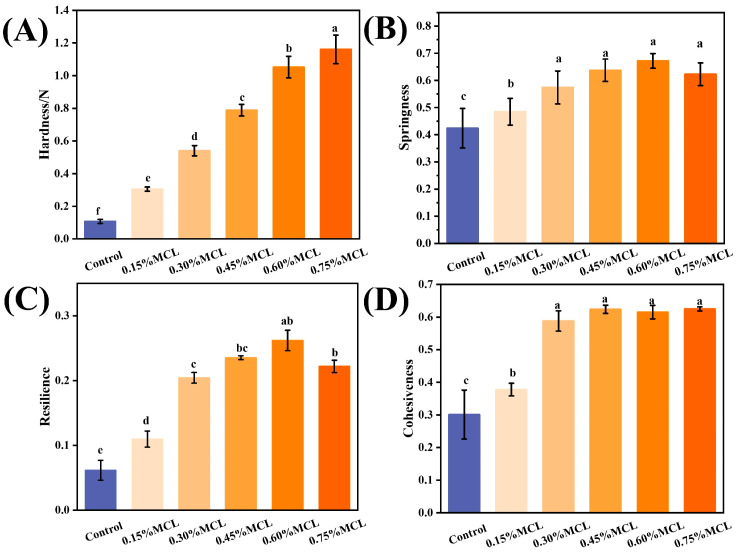
Hardness (**A**), Springiness (**B**), Resilience (**C**), and Cohesiveness (**D**) of the OBs-based oleogels. Different letters (a–f) indicate significant differences (*p* < 0.05).

Figure 6 presents the texture profile analysis (TPA) results of OBs-based oleogels, showing their hardness, cohesiveness, springiness, and resilience. As shown in Figure 6A, the hardness significantly increased (*p* < 0.05) from 0.11 N to 1.15 N with the addition of MCL. This hardening trend is attributed to enhanced intermolecular interactions among the oleogelators, which form stronger and more compact micro-network structures around the oil droplets [36]. Cohesiveness also increased significantly from 0.30 to 0.63, indicating improved structural integrity and resistance to deformation. Springiness, which reflects the ability to recover original height after force removal, increased with stronger intermolecular interactions, with the highest value (0.67) observed at 0.6% MCL. Resilience also improved (from 0.06 to 0.26), suggesting that MCL enhances the capacity for rapid shape recovery after compression.

For spreadable products (such as mayonnaise and salad dressing), spreadability is inversely related to hardness. Oleogels for spreading thus require lower hardness and moderate cohesiveness to enable smooth application without excessive adhesion [37]. In contrast, oleogels for meat analogs need higher hardness, cohesiveness, and springiness to retain structural shape and stability [38]. When replacing solid fats in baked products (such as bread, cakes, and biscuits), oleogels should maintain high springiness and resilience alongside moderate cohesiveness [39]. Future research should establish specific thresholds to optimally modulate these textural properties, which is crucial for advancing OB-based oleogels as alternatives to traditional fats.

#### 2.2.5. FTIR Analysis of Oleogels

**Figure 7:**
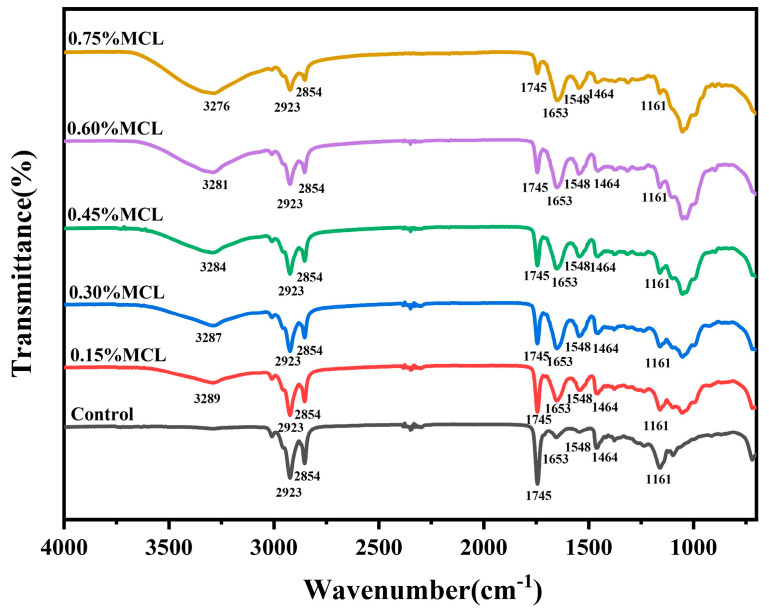
FTIR analysis of OBs-based oleogels.

The FTIR spectrum of the oleogel is shown in Figure 7. The peaks observed at 2923 cm^−1^ and 2854 cm^−1^ correspond to C-H stretching vibrations of -CH_3_ and -CH_2_ groups, respectively. The sharp absorption near 1745 cm^−1^ and the peak at 1464 cm^−1^ are attributed to C=O vibration of ester bonds and CH_2_ scissoring bending vibration, respectively [6,7,10,40]. Characteristic protein peaks from oil bodies emerge near 1563 and 1648 cm^−1^, assigned to C=O stretching (amide I band), N-H bending, and C-N stretching (amide II band) [7,12,41].

The broad peak in the 3050–3700 cm^−1^ range originates from hydroxyl groups of modified cellulose. With increasing MCL content, the peak area expanded while showing a red-shifting trend (3289–3276 cm^−1^), indicating stronger intermolecular hydrogen bonding and denser network formation at higher HF-CL concentrations, which enhances oil phase encapsulation. Notably, no new peaks emerged in OBs-based oleogels across MCL concentrations, indicating non-covalent interactions govern gel network formation [42].

## 3. Conclusions

This study utilized rubber seeds to prepare OBs-based oleogels. Through HPM modification, MCL exhibited enhanced functionality with a water-holding capacity of 29.37 g/g and an oil-holding capacity of 28.39 g/g. Oleogels prepared from OB emulsions containing ≥ 0.45% MCL exhibited good storage stability. After freeze-drying, the resulting products showed reduced surface oil leakage and a firmer structure. Furthermore, the oleogels formed by shearing demonstrated high oil binding capacity (>98.6%). With increasing MCL concentration, the textural properties were continuously improved: hardness significantly increased from 0.11 N to 1.15 N and cohesiveness from 0.30 to 0.63, while both resilience and springiness reached their peak values (0.26 and 0.67, respectively) at 0.6% MCL. These improvements are attributed to stronger intermolecular interactions and the formation of more compact micro-networks. Frequency sweeps showed significantly elevated G′ and G″ moduli, with G′ consistently dominating G″ and no crossover observed, indicating solid-like behavior. The oleogels also exhibited enhanced gel strength (G′ > 10^5^ Pa) and robust thixotropic recovery (DSR > 51%), peaking at 63.43% with 0.45% MCL. FTIR analysis further confirmed that MCL facilitates the formation of denser hydrogen-bonded networks that effectively trap the oil phase.

These findings demonstrate that MCL and OB-associated proteins synergistically form well-structured elastic networks, enabling high-performance oleogels at very low gelator concentrations. The developed oleogels show significant potential as low-fat, non-toxic alternatives to conventional fats in mayonnaise, cream, and related products.

## 4. Materials and Methods

### 4.1. Materials

Rubber seeds were purchased from Huakun Biotechnology Co., Ltd. (Xishuangbanna, China), All chemicals, including sodium hydroxide (AR grade), sucrose (AR grade), NaOH (AR grade), and sodium chlorite (80% purity), were purchased from Macklin Biochemical Technology Co., Ltd. (Shanghai, China).

### 4.2. Extraction and Modification of Cellulose from Rubber Seed Shells

Cellulose was extracted from rubber seed shells according to the method described by Freixo et al. [28] with slight modifications. Briefly, the shells were ground and sieved through a 60-mesh screen to obtain a fine powder. The powder was mixed with 6% (*w*/*v*) NaOH solution at a solid-to-liquid ratio of 1:10 (*w*/*v*) and stirred constantly at 400 rpm at 80 °C for 3 h in a water bath. For the bleaching step, the alkali-treated residue was mixed with 1.6% (*w*/*v*) NaClO_2_ solution at a ratio of 1:10 (*w*/*v*) and stirred at 400 rpm at 70 °C for 2 h. The bleaching procedure was repeated three times. After each cycle, the sample was washed thoroughly with deionized water and filtered to collect the solid residue. Finally, the purified cellulose was dried at 60 °C for 15 h and sieved through a 60-mesh sieve.

The cellulose was dispersed in distilled water to prepare a 1% (*w*/*v*) suspension. The suspension was then treated using a high-pressure microfluidizer (M-110EH-30, Micr ofluidics Corp., Westwood, MA, USA) at 80 MPa for six cycles. The samples were designated as RS (rubber seed shells), CL (cellulose isolated from the shells), and MCL (HPM-modified cellulose).

### 4.3. Extraction of OBs

OBs were extracted from rubber seeds according to the method of WANG et al. [43] with modifications. Briefly, rubber seeds were soaked in distilled water (1:5, *w*/*v*) at 4 °C for 20 h, then ground with 0.8 M sucrose solution (1:5, *w*/*v*). The homogenate was filtered through three-layer cheesecloth and centrifuged at 10,000× *g* for 20 min (4 °C). The floating OBs layer was collected, resuspended in aqueous solution, and centrifuged three times under identical conditions to remove residual sucrose. Purified OBs were stored at 4 °C for further analysis. The OB composition was as follows: protein 3.62 ± 0.14%, oil 71.21 ± 0.05%, water 23.42 ± 0.51%, and total phospholipids 1.11 ± 0.12%.

### 4.4. Preparation of OBs Emulsion and OBs-Based Oleogels

**Figure 8:**
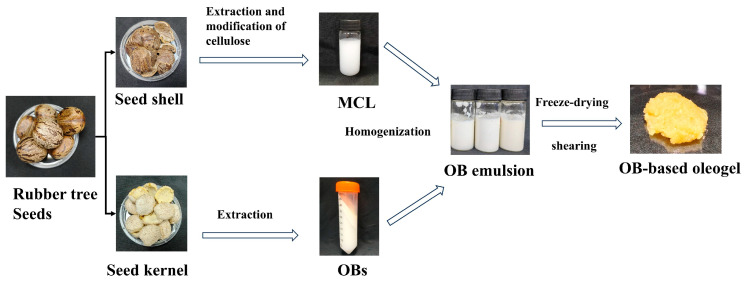
Preparation of rubber seed oil body-based oleogel.

In Figure 8 the modified cellulose solution, OBs, and distilled water were mixed to achieve an oil-to-water phase ratio of 1:3, with MCL concentrations of 0, 0.15%, 0.3%, 0.45%, 0.6%, and 0.75% (*w*/*w*). The mixture was homogenized at 10,000 rpm for 2 min to form an OBs emulsion, pre-frozen at −20 °C, and lyophilized for 24 h. The freeze-dried product was sheared at 600 rpm for 2 min to obtain MCL-modified oleosome-based oleogels. The control sample contained no MCL (designated “Control”), while MCL-containing groups were labeled 0.15–0.75%MCL.

### 4.5. Characterization of Cellulose

#### 4.5.1. Physicochemical Properties of Cellulose

Water-holding capacity (WHC) was determined in triplicate according to WANG et al. [44] with modifications. Briefly, 0.5 g of CL or MCL was hydrated with 20 mL of distilled water (40:1 *v*/*w* ratio) in a 50 mL centrifuge tube. The mixture was vortexed (30 s) and equilibrated (24 h, 25 ± 1 °C) with periodic agitation. Following centrifugation (5000× *g*, 10 min, 25 °C), the supernatant was decanted at a 45° angle (2 min). WHC was calculated as:

(1) $$WHC\left(\frac{g}{g}\right)\;=\;\frac{W_{2}}{W_{1}}\;\times \;100\%$$

where *W*_2_ and *W*_1_ are the weights of water adsorbed and the CL or MCL sample, respectively.

Oil-holding capacity (OHC) was determined in triplicate according to Xie et al. [15]. Briefly, 0.5 g of CL or MCL was combined with 5 mL of edible oil in a pre-weighed 15 mL centrifuge tube. After vortex mixing (1 min), the suspension was equilibrated (10 h, 25 °C) without agitation. Centrifugation was performed at 6000× *g* (15 min, 25 °C). The supernatant was decanted at 45° for 5 min, and the tube with adsorbed oil was weighed. OHC was calculated as:

(2) $$OHC\left(\frac{g}{g}\right)\;=\;\frac{W_{3}}{W_{4}}\;\times \;100$$

where *W*_3_ and *W*_4_ are the weights of oil adsorbed and the CL or MCL sample, respectively.

Swelling capacity (SC) was determined in triplicate according to Xie et al. [15] with modifications. Briefly, 0.2 g of CL or MCL (dry weight) was accurately weighed into a 10 mL graduated cylinder. After adding 5 mL of distilled water, the mixture was vortexed (30 s) and hydrated (18 h, 25 °C). Following sedimentation (1 h), the swollen volume was recorded. SC was calculated as:

(3) $$SC\left(\frac{mL}{g}\right)\;=\;\frac{V}{W}\;\times \;100\%$$

where *V* is the final volume occupied by CL or MCL, and *W* is the weight of CL or MCL.

#### 4.5.2. FTIR Analysis of Cellulose

The FTIR spectra of the samples were obtained using an FTIR spectrometer (Nicolet IS50, Thermo Fisher Scientific, Madison, WI, USA). The sample was ground and mixed with KBr, which was immediately pressed into a tablet under a pressure of 30 MPa. The spectral range was 4000–400 cm^−1^ with a resolution of 4 cm^−1^.

#### 4.5.3. Scanning Electron Microscopic

SEM images of CL and MCL samples were captured using a scanning electron microscope (S-4800, Hitachi High-tech, Tokyo, Japan). To facilitate observation, the sample was coated with a layer of gold before being examined, and they were observed under an accelerating voltage of 10 kV. The micromorphologies of CL and MCL samples were observed at ×100 and ×500 magnifications.

#### 4.5.4. X-Ray Diffraction

X-ray diffraction (XRD) patterns of CL and MCL were acquired using a diffractometer (X’pert Powder, PANalytical, Almelo, The Netherlands). Scans were performed at 40 kV and 40 mA over a 2θ range of 5° to 40° with a scanning rate of 2°/min^−1^. The crystallinity index (CrI) was calculated according to the following equation:

(4) $$CrI\left(\%\right)\;=\;\frac{I_{200}\;-{\;I}_{AM}}{I_{200}}\;\times \;100\%$$

where *I*_200_ refers to the diffraction intensity of the (200) plane, which corresponds to the crystalline region as well as the amorphous region. *I_AM_* represents the minimum intensity observed between the (200) and (110) planes, representing the amorphous region.

#### 4.5.5. Brunauer–Emmett–Teller (BET) Analysis

The surface areas of CL and MCL were analyzed using the BET analyzer Tristar II Plus (Micrometrics, Norcross, GA, USA). The degassing temperature and time were 30 °C and 15 h, respectively.

### 4.6. Characterization of Different Oleogels

#### 4.6.1. Oil Binding Capacity of Oleogels

The oil-holding capacity (OHC) of OBs-based oleogels was determined according to [10,24]. Approximately 1 g of oleogel was placed in a 10 mL centrifuge tube and centrifuged at 10,000 rpm for 10 min. The tube was then inverted to drain the expelled oil, and residual oil on the tube wall was carefully removed with cotton swabs. The OHC was calculated using the following formula:

(5) $$OHC\left(\%\right)\;=\;\frac{m_{1}\;-\;m_{2}}{m_{1}\;-\;m_{0}}\;\times \;100\%$$

where *m*_0_, *m*_1_, and *m*_2_ are the masses of the empty tube, tube with OBs-based oleogels, and tube with processed oleogel, respectively.

#### 4.6.2. FTIR Analysis of Oleogels

FTIR spectra of OBs-based oleogels were acquired using an in ATR mode. Spectra were collected over the range of 400 to 4000 cm^−1^ with 32 scans at a resolution of 4 cm^−1^.

#### 4.6.3. Rheological Properties of Oleogels

The rheological properties of OBs-based oleogels were characterized using a rotational rheometer (Haake MARS 60, Thermo Fisher Scientific, Madison, WI, USA) The setup consists of a 50 mm parallel plate geometry (PP35), and a 1 mm gap was fixed for all experiments.

Linear viscoelastic region (LVR) determination: At a fixed frequency of 1 Hz and controlled temperature of 25.0 ± 0.1 °C, strain amplitude sweeps (0.1% to 50%) were performed to identify the LVR for subsequent frequency tests. Frequency-dependent viscoelasticity: Within the LVR (0.5% strain), frequency sweeps from 0.1 to 10 Hz were conducted to analyze the frequency dependence of storage modulus (G′) and loss modulus (G″). Thermal responsiveness: At constant stress and frequency (1 Hz), temperature sweeps from 25 °C to 80 °C were performed at a heating rate of 5 °C/min, monitoring real-time viscosity evolution. Structural recovery analysis: Samples were sheared at 0.1 s^−1^ for 200 s, followed by 10 s^−1^ for 120 s, and then at 0.1 s^−1^ for 200 s. Viscosity changes were recorded throughout. The degree of structure recovery (DSR) of the oleogels was calculated by dividing the viscosity values obtained during the 3rd to 1st step.

#### 4.6.4. Texture Profile Analysis

Cylindrical oleogel samples were prepared in PTFE molds and conditioned at 25 °C for 12 h. Texture Profile Analysis (TPA) was performed using a texture analyzer (TA.XTPlus, Stable Micro Systems, Godalming, UK) with a 60° conical probe (P/6). Settings: Trigger: 0.05 N (5 g load cell), Pre-test: 1.0 mm/s, Compression: 50% strain (45 mm displacement), Retraction: 4.0 mm/s.

#### 4.6.5. CLSM Analysis

CLSM images of the samples were obtained on a microscope (LSM900, Carl Zeiss, Jena, Germany) with a magnification of 40×. Before imaging, the oleogels were stained with FITC (0.10%, *w*/*v*) to visualize the proteins. The incident laser wavelength was set at 488 nm for FITC excitation.

### 4.7. Statistical Analysis

Data are presented as mean ± standard deviation. Statistical analyses, including ANOVA to assess significant differences, were executed with the SPSS 26.0 software, with a *p*-value ≤ 0.05 indicating statistical significance. Each experiment was repeated at least three times.

## Figures and Tables

**Table 1:** Basic properties of CL and MCL.

Sample	WHC (g/g)	OHC (g/g)	SC (mL/g)
CL	4.92 ± 0.11 ^b^	7.41 ± 0.14 ^b^	0.65 ± 0.01 ^b^
MCL	29.37 ± 0.93 ^a^	28.39 ± 1.01 ^a^	3.38 ± 0.80 ^a^

Different letters (a, b) indicate significant differences (*p* < 0.05). WHC water-holding capacity, OHC oil-holding capacity, SC swelling capability.

## Data Availability

The original contributions presented in the study are included in the article; further inquiries can be directed to the corresponding author.

## Supplementary Materials

The following supporting information can be downloaded at: https://www.mdpi.com/article/10.3390/gels11100819/s1, Table S1. Surface area of CL and MCL; Figure S1. CLSM of the OBs-based oleogels.
