# Supplementary material for: Preparation and Characterization of Oleogels Based on Cellulose Modified by High-Pressure Microfluidization and Rubber Seed Oil Body
Source: Gels. 2025 Oct 13;11(10):819. doi: 10.3390/gels11100819 (PMC12564632; doi:10.3390/gels11100819)
Supplement: Supplementary file 1 [file gels-11-00819-s001.zip › Supplementary Files.pdf]

Table S1. Surface area of CL and MCL.

Sample	Surface area (m^2/g)
CL	0.92 ± 0.18^{c}
MCL	6.47 ± 0.11^{a}

Different letters (a, b) indicate significant differences ($p < 0.05$).

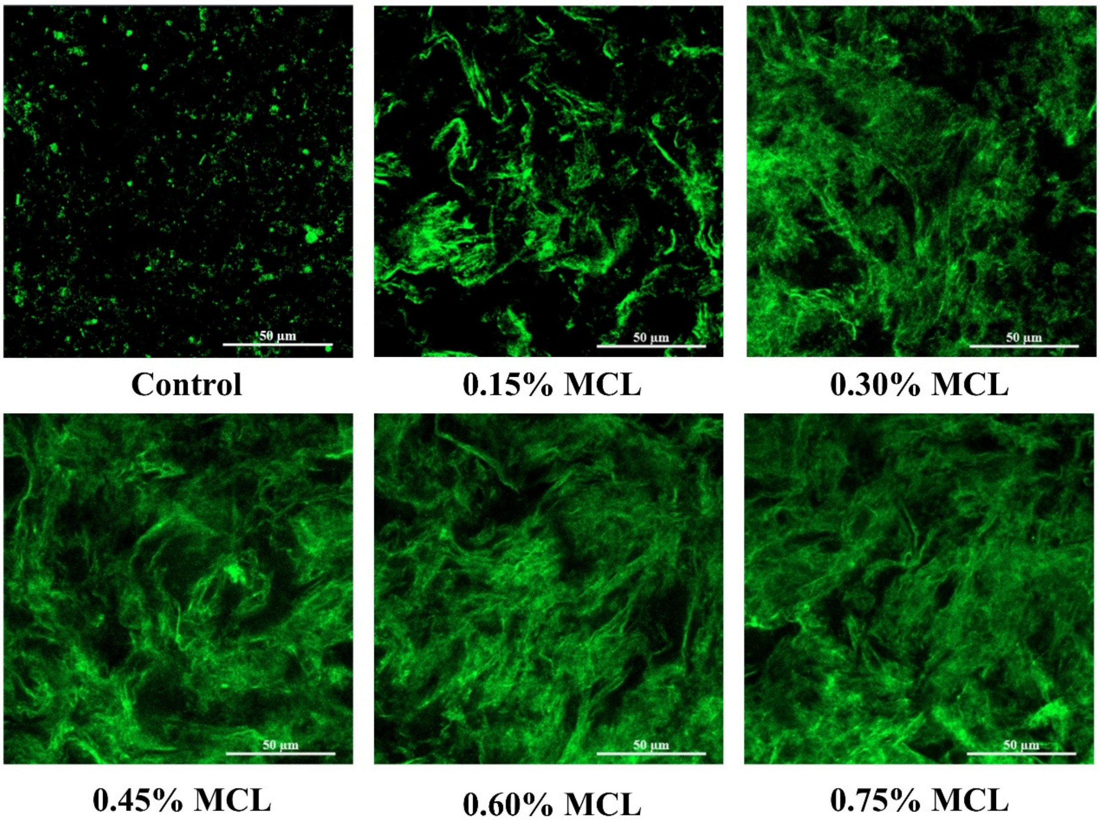

Figure S1. CLSM of the OBs-based oleogels.
