# Supplementary figures and images for: Preparation and Characterization of Oleogels Based on Cellulose Modified by High-Pressure Microfluidization and Rubber Seed Oil Body
Source: Gels. 2025 Oct 13;11(10):819. doi: 10.3390/gels11100819 (PMC12564632; doi:10.3390/gels11100819)

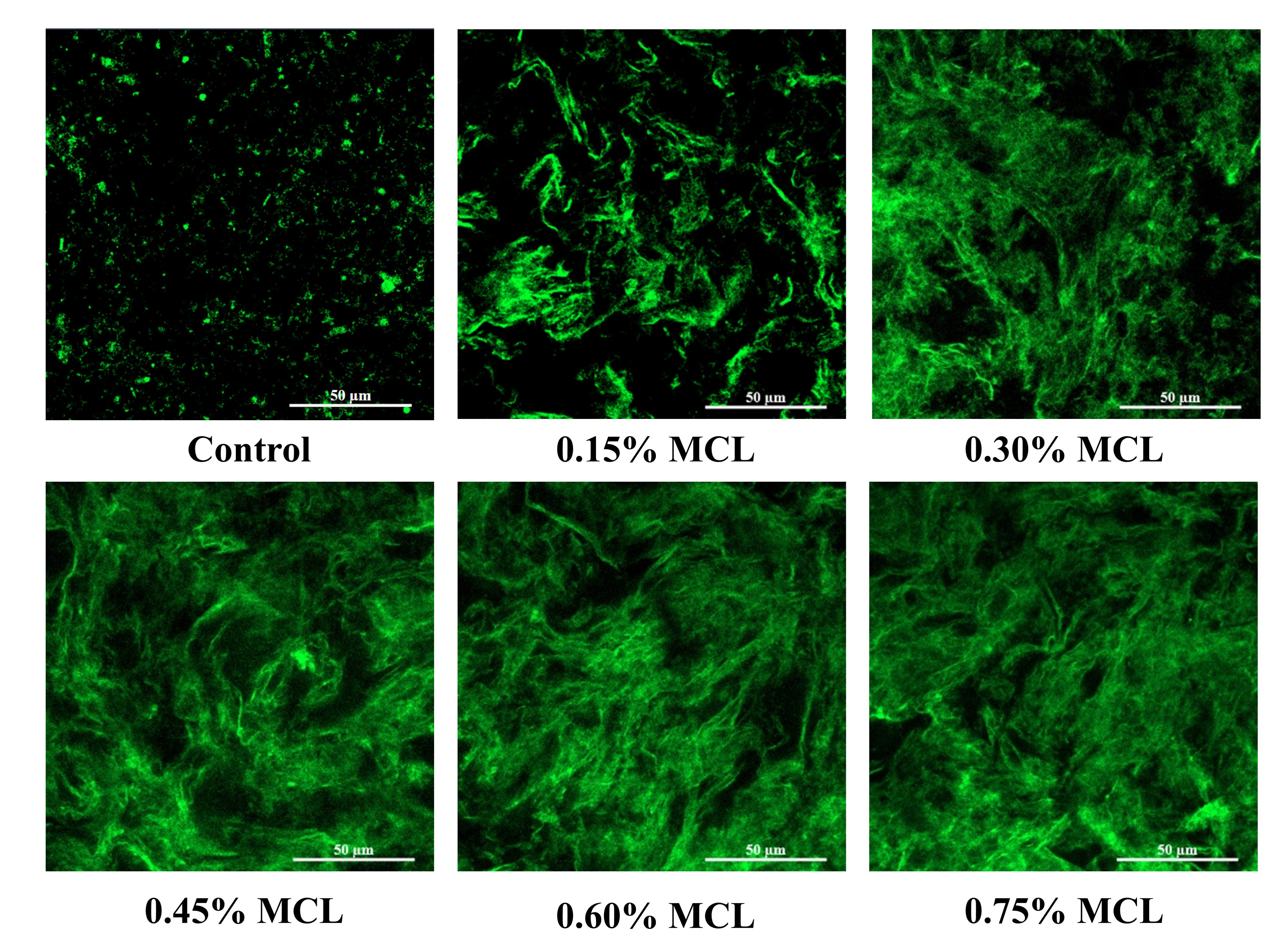

Supplement: Supplementary file 1 [file gels-11-00819-s001.zip › Figure S1.jpg]
